# Supplementary material for: Efficient Flexible All-Solid Supercapacitors with Direct Sputter-Grown Needle-Like Mn/MnOx@Graphite-Foil Electrodes and PPC-Embedded Ionic Electrolytes
Source: Nanomaterials (Basel). 2020 Sep 7;10(9):1768. doi: 10.3390/nano10091768 (PMC7557606; doi:10.3390/nano10091768)
Supplement: Supplementary file 1 [file nanomaterials-10-01768-s001.pdf]

Supplementary File

Efficient Flexible All-Solid Supercapacitors with Direct Sputter-Grown Needle-Like Mn/MnOx@Graphite-Foil Electrodes and PPC-Embedded Ionic Electrolytes

Apurba Ray [1], Delale Korkut [1,2] and Bilge Saruhan [1,*]

[1] Department of High-Temperature and Functional Coatings, Institute of Materials Research, German Aerospace Center (DLR), 51147 Cologne, Germany; e-mail: apurba.ray@dlr.de

[2] Department of Chemistry, RWTH Aachen University, 52062 Aachen, Germany; e-mail: delale.korkut@dlr.de

* Correspondence: bilge.saruhan@dlr.de

Table 1. Comparison of supercapacitive performance of various MnO_x–carbon composites electrodes based supercapacitors (SC) in literature and the present work.

Electrode Material	Electrolyte	Type of SCs	Stable potential window	Maximum specific capacitance	Maximum energy density	Maximum power density	Ref.
MnO_2@CNT	1 M Na_2SO_4 aqueous electrolyte	ASC	2.0 V	61 F/g @ 0.2 A/g	27.8 Wh/kg	10000 W/kg	[1]
MnO_2 nanosheets on flexible carbon foam.	1.0 M Na_2SO_4 aqueous electrolyte	SC	0.7 V	1270.5 F/g @ 0.5 A/g	86.2 Wh/ kg	174.8 W /kg	[2]
Mn_3O_4 Nanoflakes on Carbon Fibers.	1 M Na_2SO_4 aqueous electrolyte	ASC	1.8 V	65 F/g @ 0.5 A/g	14 Wh/ kg	9000 W /kg	[3]
MnO_2/CNT/ papers.	0.1 M Na_2SO_4 aqueous electrolyte	SC	0.8 V	540 F/g @ 5.0 A/g	20 Wh/ kg	1.5 kW /kg	[4]
manganese oxide nanosheet/carbon cloth.	0.1 M Na_2SO_4 aqueous electrolyte	SC	0.9 V	230 mF/ cm^2 @ 0.13 mA/cm^2	-----	-----	[5]
MnO_2 – Graphene.	1 M Na_2SO_4 aqueous electrolyte	ASC	1.8 V	69.4 F/g @ 0.5 A/g	31.8 Wh/ kg	9188.1 W/ kg	[6]
MnO_2-Graphene.	1 M KCl aqueous electrolyte	ASC	1.0 V	328 F/g	11.4 Wh/kg	25.8 kW/kg	[7]
CNTs/MnO_2	1 M Na_2SO_4 aqueous electrolyte	ASC	2.0 V	152 F/g @ 0.3 A/g	84.6 W h/kg	4748 W/kg	[8]
**Mn/MnO_x@ Graphite-foil**	**[EMIM][TFSI]:PCC=1**	**SC**	**2.2 V**	**11.71 mF/cm^2@ 0.03 mA/cm^2**	**7.87 mWh/cm^2**	**1099.64 mW/cm^2**	**This work**

ASC = asymmetric supercapacitors; SC= symmetric supercapacitor;

Reference

[1] Lee, T. H.; Pham, D. T.; Sahoo, R.; Seok, J.; Luu, T. H. T.; Lee,Y. H. High energy density and enhanced stability of asymmetric supercapacitors with mesoporous MnO_2@CNT and nanodot MoO_3@CNT free-standing films. Energy Storage Mater. 2018, 12, 223–231.

[2] He, S.; Chen ,W. High performance supercapacitors based on three-dimensional ultralight flexible manganese oxide nanosheets/carbon foam composites. *J. Power Sources.* 2014, 262, 391–400.

[3] Rafique, A.; Zubai, U.; Serrapede, M.; Fontana, M.; Bianco, S.; Rivolo, P.; Pirri, C. F.; Lamberti, A. Binder Free and Flexible Asymmetric Supercapacitor Exploiting Mn_3O_4 and MoS_2 Nanoflakes on Carbon Fibers. *Nanomaterials*, 2020, 10, 1084.

[4] Kang, Y. J.; Kim, B. w.; Chung, H.; Kim,W. Fabrication and characterization of flexible and high capacitance supercapacitors based on MnO_2/CNT/papers. *Synth. Met.* 2010, 160, 2510–2514.

[5] Chen,Y.-C.; Hsu, Y.-K.; Lin, Y.-G.; Lin, Y.-K.; Horng, Y.-Y.; Chen, L.-C.; Chen, K.-H. Highly flexible supercapacitors with manganese oxide nanosheet/carbon cloth electrode. *Electrochim. Acta* 2011, 56, 7124–7130.

[6] Zhang , Z.; Xiao, F.; Qian, L.; Xiao, J.; Wang, S.; Liu , Y. Facile Synthesis of 3D MnO_2 –Graphene and Carbon Nanotube–Graphene Composite Networks for High-Performance, Flexible, All-Solid-State Asymmetric Supercapacitors. *Adv. Energy Mater.* 2014, 4, 1400064.

[7] Q. Cheng.; J. Tang.; J. Ma.; H. Zhang.; N. Shinya.; L.C. Qin. Graphene and nanostructured MnO_2 composite electrodes for supercapacitors. *Carbon.* 2011, 49, 2917 – 2925.

[8] Jia, H.; Cai, Y.; Zheng, X.; Lin, J.; Liang, H.; Qi, J.; Cao, J.; Feng, J.; Fei, W. Mesostructured Carbon Nanotube-on-MnO_2 Nanosheet Composite for High-Performance Supercapacitors. *ACS Appl. Mater. Interfaces.* 2018, 10, 38963−38969.
